# Sponge-like Chitosan Based Porous Monolith for Uraemic Toxins Sorption

**DOI:** 10.3390/nano11092247

**Published:** 2021-08-30

**Authors:** Siyu Xiong, Yaxuan Lyu, Andrew Davenport, Kwang Leong Choy

**Affiliations:** 1 UCL Institute for Materials Discovery, University College London, London WC1E 7JE, UK; siyu.xiong.15@ucl.ac.uk; 2 MRC Laboratory for Molecular Cell Biology, University College London, London WC1E 6BT, UK; yaxuan.lyu.17@ucl.ac.uk; 3 UCL Centre for Nephrology, Royal Free Hospital, University College London, London NW3 2PF, UK; andrewdavenport@nhs.net

**Keywords:** sponge-like porous materials, absorbent courier, uraemic toxin removal, chitosan, casting

## Abstract

More than three million patients are treated for kidney failure world-wide. Haemodialysis, the most commonly used treatment, requires large amounts of water and generates mountains of non-recyclable plastic waste. To improve the environmental footprint, dialysis treatments need to develop absorbents to regenerate the waste dialysate. Whereas conventional dialysis clears water-soluble toxins, it is not so effective in clearing protein-bound uraemic toxins (PBUTs), such as indoxyl sulfate (IS). Thus, developing absorption devices to remove both water-soluble toxins and PBUTs would be advantageous. Vapour induced phase separation (VIPS) has been used in this work to produce polycaprolactone/chitosan (PCL/CS) composite symmetric porous monoliths with extra porous carbon additives to increase creatinine and albumin-bound IS absorption. Moreover, these easy-to-fabricate porous monoliths can be formed into the required geometry. The PCL/CS porous monoliths absorbed 436 μg/g of albumin-bound IS and 2865 μg/g of creatinine in a single-pass perfusion model within 1 h. This porous PCL/CS monolith could potentially be used to absorb uraemic toxins, including PBUTs, and thus allow the regeneration of waste dialysate and the development of a new generation of environmentally sustainable dialysis treatments, including wearable devices.

## 1. Introduction

World-wide more than three million patients with chronic kidney disease (CKD) receive renal replacement therapy, with the great majority treated by haemodialysis (HD). Whereas the HD is very effective in clearing small water-soluble uraemic toxins, the HD is much less effective in clearing protein-bound uraemic toxins (PBUTs) [1], with reported clearances of the PBUT indoxyl sulfate (IS) of 10–15 mL/min compared to a urea clearance of 200 mL/min. Thus, PBUTs accumulate with progressive CKD [1,2,3] and have been observed to increase the risk of cardiovascular disease [4,5,6,7]. As conventional dialysers do not effectively remove these PBUTs, newer biomaterials and designs are required.

Traditional HD requires large volumes of water to produce fresh dialysate for single-pass treatments and generates vast quantities of non-recyclable plastic waste for landfills. The introduction of adsorbents would potentially both increase PBUT clearances and also permit the regeneration and recycling of waste dialysate, allowing the development of wearable dialysis devices.

There are two main approaches for the design of uraemic toxin sorbents: (1) physical adsorption from highly porous materials; and (2) non-physical interactions (e.g., chemical bonding and conjugation effects) between targeted uraemic toxins and absorptive materials. Currently, porous materials, such as zeolite [8], mesoporous carbon [9,10], and metal organic frameworks (MOFs) [11], are popular absorbent choices to remove uraemic toxins, due to their high adsorption performance to bind PBUTs (i.e., via the physical adsorption). Meanwhile, chitosan, a non-porous polysaccharide used in water purification [12,13], was found to reduce the indoxyl sulfate concentration in aqueous solution via the non-physical interactions between chitosan and indoxyl sulfate [14].

Although these putative uraemic solute absorbents have been studied, how best to deliver the absorptive materials and stabilise them in dialysers or cartridges to allow for effective blood contact remains to be determined. The supportive material used is important for both sorbent immobilisation and will determine the effective surface area for contact with uraemic toxins. Electrospun fibres [8,14] and carbonised monoliths [9,15] have been explored, but were found to have technical problems with the fabrication process and limited contact with PBUTs. The ideal supportive material would be an easy-to-fabricate, cost-effective water permeable porous sorbent structure with compatibility to multiple absorptive/adsorptive compounds.

Vapour induced phase separation (VIPS) is a well-established fabrication strategy for porous membranes [16,17] and electrospun fibres [18], which utilises the water vapour to trigger the phase separation process and create symmetrically distributed internal and external micro pores in the hydrophobic polymeric structure [19]. The VIPS approach can allow casting at room temperature and atmosphere, thus reducing costs, and this approach is also compatible with combining multiple additives, thereby providing composites with multiple functions.

We report the fabrication of a polycaprolactone/chitosan (PCL/CS) composite based on VIPS mechanism to form a sponge-like PCL/CS porous monolith as a single-use absorbent for uraemic toxin sorption. The absorptive capacity with creatinine and albumin-bound IS as a multiple absorptive materials courier for creatinine and albumin-bound IS absorption has been tested as a proof-of-concept. Porous carbon was incorporated into the PCL/CS monolith to evaluate the feasibility of multi-additives into this monolith.

## 2. Materials and Methods

### 2.1. Preparation of PCL/CS Porous Monolith Casting Solutions

Anhydrous dimethylformamide (DMF) (Analytical reagent grade, ≥99.5%, D/3841/17, Fisher Scientific, Loughborough, UK) and formic acid (FA) (ACS reagent, 33015-1L-M, Sigma-Aldrich, Gillingham, UK) were mixed in a 7/3 volume ratio (Table 1). Chitosan (CS) (deacetylation = 80.0~95.0%, 69047436-100G, Sinopharm Chemical Reagent Co., Ltd., Shanghai, China) powder and polycaprolactone (PCL) (Mn = 80,000, 440744-125G, Sigma-Aldrich, Gillingham, UK) were mixed and dissolved into the DMF/FA solvent in a glass vial, and stirred at 40 °C for 24 h, forming the homogenous PCL/CS casting solutions. When the PCL and CS were completely dissolved, additive powders (graphene nanoplatelets (25μm particle size, surface area 120–150 m^2^/g, 900413-100G, Aldrich, Gillingham, UK) and NORIT (E Supra USP, 8030-7, CABOT Corporation, Alpharetta, GA, USA) were added into the homogenous PCL/CS casting solutions and stirred for a further 15 min prior to casting. Details of the casting solutions are listed in Table 1.

### 2.2. Preparation of the PCL/CS Porous Monolith

**Figure 1:**
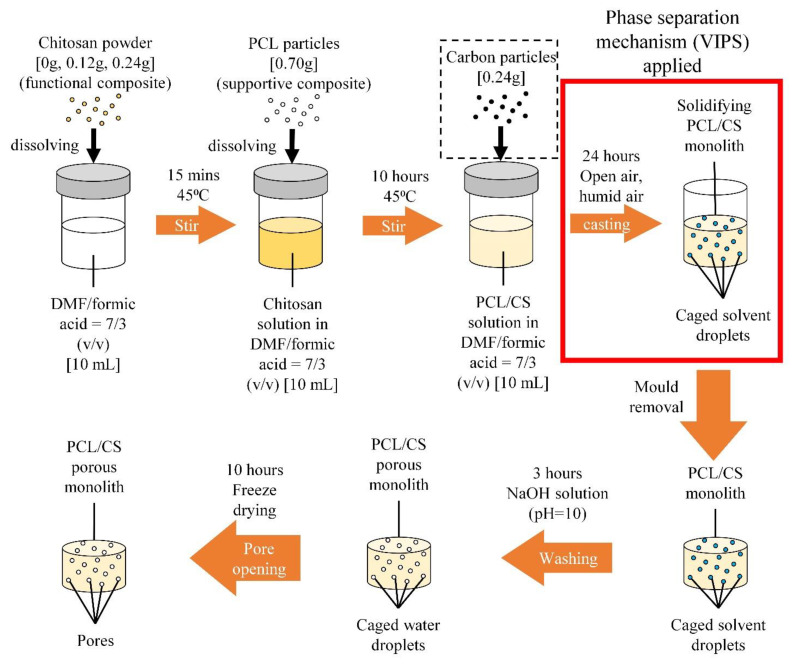
The fabrication steps of PCL/CS porous monoliths.

The casting solutions were poured into removable 2 mL cylindrical casting moulds in a fume hood. The casting process took 24 h at room temperature and atmosphere until the monoliths were completely solidified, and the surfaces were porous and dry. The solidified PCL/CS porous monoliths were then de-moulded and washed with NaOH aqueous solution (pH = 10) for 3 h to further coagulate the polymer and replace the DMF and formic acid residuals with water for opening pores. The washed PCL/CS porous monoliths were finally freeze dried at −51 °C and 0.024 mbar to sublime the ice, forming dried PCL/CS porous monolith (cylindrical shape). These cylindrical PCL/CS porous monoliths were then cut into cuboids with an average dimension of 8.0 × 10.0 × 4.0 mm (width × length × height) and weighed prior to absorption testing. The overview of the PCL/CS porous monolith fabrication process is illustrated in Figure 1.

### 2.3. Surface Morphology

The PCL/CS porous monoliths were then cut into slices and gold-coated (Q 150R ES, Quorum Technologies Ltd., Laughton, UK) to a depth of 15 nm before characterisation by scanning electron microscopy (SEM) (EVO LS15, Carl Zeiss AG, Jena, Germany) to determine their surface morphology.

### 2.4. Composition Validation by TGA and FTIR

The composition of the PCL/CS porous monoliths was characterised using a combination of a Fourier-transform infrared spectroscopy (FTIR) (Spectrum Two, PerkinElmer, Beaconsfield, UK) and a thermogravimetric analyser (TGA) (TGA 4000, PerkinElmer, Beaconsfield, UK). The FTIR spectra of the PCL/CS porous monoliths were obtained from a 5-min scan ranging from 4000 cm^−1^ to 400 cm^−1^ wavenumbers with a 2.0 cm^−1^ resolution. Absorption peaks of the hydroxyl and amine groups were used to identify the chitosan content. The TGA test was conducted and compared among: (1) pure PCL; (2) pure chitosan; and (3) PCL/CS porous monoliths, with a test temperature ranging from 30 °C to 500 °C. The sample temperature was increased from 30 °C to 100 °C rapidly at 35 °C/min, then held at 100 °C for 15 min to remove all water and oxygen. The temperature was then increased from 100 °C to 500 °C at 10 °C/min. Nitrogen was used with a gas flow rate of 20 mL/min. The three materials were tested under the same conditions in order to obtain comparable TGA curves.

### 2.5. Porosity Analysis by BET Method

The specific surface area of the porous monoliths was measured using Brunauer–Emmett–Teller (BET) equipment (NOVA Touch, Quantachrome Instruments, Boynton Beach, FL, USA). The sample was trimmed into small pieces and dried under vacuum at 30 °C for 20 h and then weighed. The BET analysis was then conducted using nitrogen as the adsorbate gas at a bath temperature of 77.35 K. The bath thermal delay was set to 600 s with helium backfill mode. The adsorption isotherm (BET plot) was obtained for the calculation of the specific surface area of the monoliths. The half pore width distribution was modelled by the density-functional theory (DFT) method.

### 2.6. Uraemic Toxin Absorption Performance Evaluation

#### 2.6.1. Albumin-Bound Indoxyl Sulfate (IS) Solution Preparation

First, 250 mg IS potassium salt (for HPLC, ≥99%, I3875-1G, Sigma-Aldrich, Gillingham, UK) was dissolved into the simulated body fluid (SBF, Xi’an Hat Biotechnology Co. Ltd., Xi’an, China) to make a 50 mL concentrated solution (IS concentration = 5 g/L) in a Falcon tube (50 mL, High-clarity polypropylene conical tube, Corning Science, Reynosa, Mexico). Then, 2.5 g human serum albumin (HSA Fraction V, MP Biomedicals, Irvine, CA, USA) was added into a 50 mL Falcon tube, and a 0.4 mL concentrated IS solution (IS concentration = 5 g/L) was subsequently added into the Falcon tube with HSA powder. Subsequently, more SBF was added to make a 50 mL solution. This HSA-IS solution was then incubated at 37 °C for 24 h. After incubation, the solution was stored in the fridge at 4 °C, ready for testing as the HSA-IS precursor solution (total IS concentration = 40 mg/L, total HSA concentration = 50 g/L).

#### 2.6.2. Creatinine Solution Preparation

First, 40 mg creatinine (anhydrous, ≥98%, Sigma-Aldrich, C4255-10G) was dissolved into 10 mL simulated body fluid (SBF, Xi’an Hat Biotechnology Co. Ltd., Xi’an, China) to make a concentrated creatinine solution (creatinine concentration = 4 g/L) in a Falcon tube. About 38.8 mL SBF was subsequently added into 0.2 mL concentrated creatinine solution to make a 40 mL solution. After vortex, the solution was ready for testing as the creatinine precursor solution (creatinine concentration = 20 mg/L).

#### 2.6.3. Test Protocol

**Figure 2:**
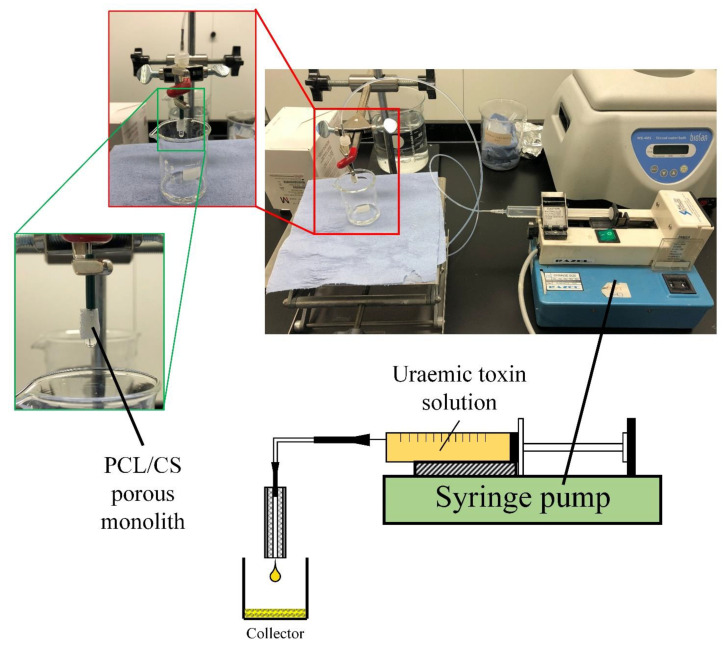
The uraemic toxins absorption test setup for the PCL/CS porous monoliths.

The uraemic toxin absorption performance of these PCL/CS porous monoliths were evaluated using the single-pass system as shown in Figure 2. The PCL/CS porous monolith was firstly connected onto the uraemic toxin precursor solution supply nozzle tip by inserting the nozzle into the monolith with a depth of 2 mm. The uraemic toxin precursor solution (prepared in Section 2.6.1 and Section 2.6.2) was pumped through the fixed PCL/CS porous monolith by using a syringe pump and collected by a glass vial for 1 h. During the test, the flow rate of the uraemic toxin precursor solution was controlled at 5.95 mL/h. The uraemic toxin concentrations in the filtered solution after passing through the absorbents fabricated under different conditions were compared with the initial uraemic toxin concentrations. The uraemic toxins absorption test setup for the PCL/CS porous monolith is illustrated in Figure 2.

### 2.7. Uraemic Toxin Concentration Measurement

To measure uraemic toxins (IS and creatinine) concentration in the sample solutions, 10 mL ice-cold acetonitrile was added to 5 mL of the sample solution to precipitate all protein content completely. Precipitated solutions were vortexed and centrifuged at 3000 rpm 25 °C for 10 min. Supernatants were then extracted for the uraemic toxins concentration measurement using a high performance liquid chromatography (HPLC) (Flexar, PerkinElmer, Beaconsfield, UK) equipped with a C18 column (Hypersil, 3 µm C18, 130 Å, LC Column 250 × 4.6 mm, Thermo Scientific, Waltham, MA, USA). Mobile phase A (66%): acetonitrile, and mobile phase B (34%): de-ionized water, were pumped through the column with a flow rate of 1.0 mL/min between 0–8 min. The injection volume of sample solutions was 20 µL. The IS and creatinine were detected at 278 nm and 234 nm, respectively, using a UV-vis detector. The absorption peaks of IS and creatinine were identified at 0.9 min and 1.0 min, respectively.

The unit uraemic toxin absorbability of the PCL/CS porous monoliths was calculated based on the weight and volume aspects by using the following equations:

(1) $$\mathrm{Unit}\;\mathrm{weight}\;\mathrm{absorbability}\;=\;\frac{\left(C_{0}-C_{F}\right)\;\times \;V_{F}}{m_{a}}$$

(2) $$\mathrm{Unit}\;\mathrm{volume}\;\mathrm{absorbability}\;=\;\frac{\left(C_{0}-C_{F}\right)\;\times \;V_{F}}{V_{a}}$$

where $C_{F}$ (mg/L) and $C_{0}$ (mg/L) were the uraemic toxin concentrations in post filtered solution and the initial unfiltered uraemic toxin solutions, respectively; $V_{F}$ (mL) was the volume of filtered uraemic toxin solution; $m_{a}$ (g) and $V_{a}$ (cm^3^) was the weight and volume of the tested PCL/CS monolith.

### 2.8. Statistics

The significance of the uraemic toxins absorption test results from the PCL/CS composite sponge-like absorbents were analysed and validated using the one-way ANOVA test in Origin (Origin Pro 2017, OriginLab Corporation, Northampton, MA, USA). Statistical difference was taken at a *p*-value of less than 0.05.

## 3. Results

### 3.1. PCL/CS Porous Monolith Demonstration and Morphology Analysis

**Figure 3:**
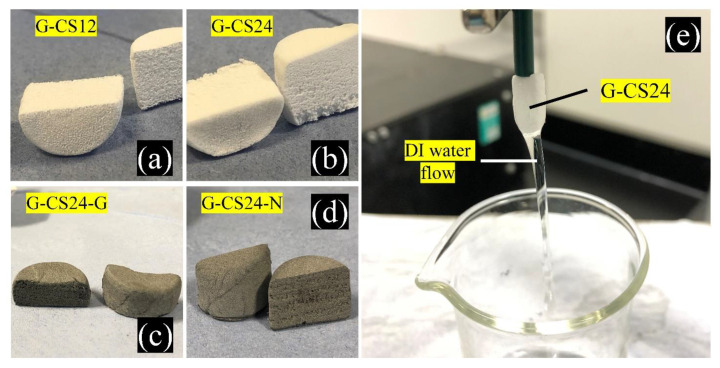
The appearance of the PCL/CS porous monoliths: (**a**) G-CS12; (**b**) G-CS24; (**c**) G-CS24-G; (**d**) G-CS24-N; and (**e**) the water flushing on the G-CS24 monolith.

The PCL/CS porous monoliths had a soft, geometrically tailorable, and sponge-like porous structure (Figure 3). Due to its internal porosity, the PCL/CS porous monoliths were permeable for aqueous solutions (Figure 3e). The IS solution was perfusing through the PCL/CS composite monolith, ensuring excellent contact between chitosan, carbon, and IS.

The G-CS0 ratio had difficulties in dissolving in the DMF/formic acid solvent, resulting in no monolith formation, while the other composites (G-CS12, G-CS24, G-CS24-G, and G-CS24-N) successfully formed porous PCL/CS composite monoliths. The cross-sectional view of the PCL/CS porous monoliths demonstrated a uniform and symmetric distribution of both internal and external pores. The G-CS24 samples had less uniform pore distribution than the G-CS12 samples and there were some visible cracks at the bottom of the monolith.

**Figure 4:**
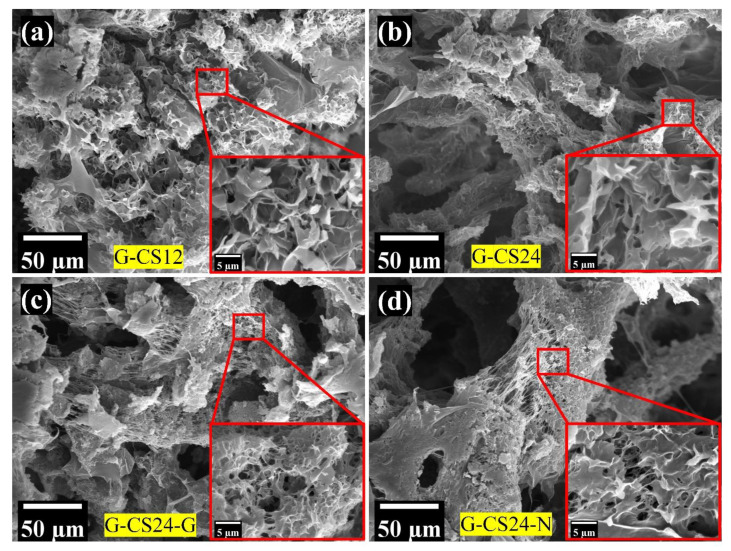
The microstructure of the PCL/CS porous monoliths: (**a**) G-CS12; (**b**) G-CS24; (**c**) G-CS24-G; (**d**) G-CS24-N.

A porous structure was observed in the SEM images (Figure 4), which indicated that the porosity of these monoliths came from the folding of cloth-like polymer layers, while the surface of these cloth-like polymer layers was still smooth. The space sandwiched between these polymer layers would allow liquid perfusion, providing greater contact between the absorptive materials and the target toxins.

**Figure 5:**
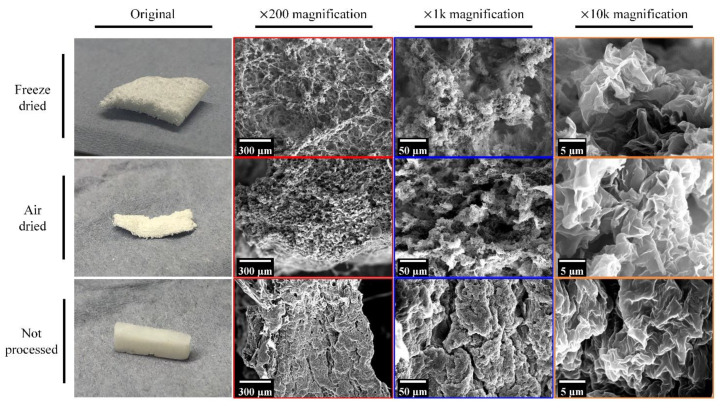
The influence of different drying process on the microstructure of the PCL/CS porous monoliths (G-CS12).

Different drying approaches were attempted and resulted in a different microstructure of the PCL/CS porous monoliths (Figure 5). The air dry and freeze dry approaches produced similar porous structure in microscale, but the freeze-dried PCL/CS porous monolith had a fluffier porous configuration than the air dried porous monolith due to the support from solid ice, which potentially reduced the difficulties of water perfusion in such a bulk structure. If the solvent in the PCL/CS monolith was not washed out or dried completely, the pores would not be formed.

### 3.2. Composition Validation

**Figure 6:**
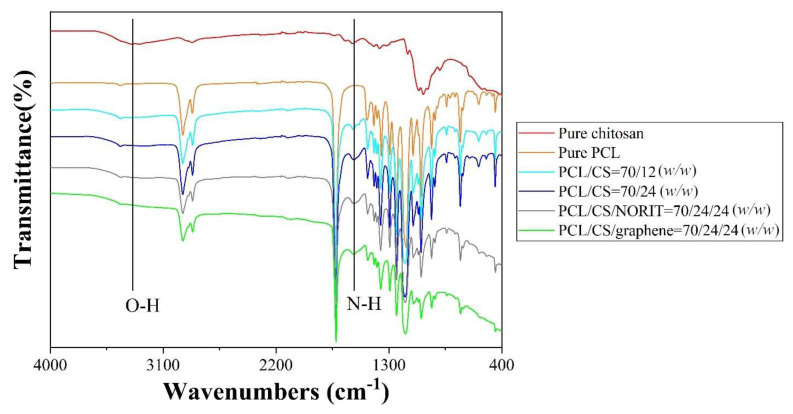
The FTIR spectra of the PCL/CS porous monoliths.

The FTIR and TGA tests were compared among all PCL/CS porous monoliths as a validation of the composition of chitosan and carbon content. In the FTIR spectra (Figure 6), an absorption peak at 1580 cm^−1^ can be observed in all PCL/CS porous monoliths and not in the pure PCL control group, which confirms the composition of chitosan content. The carbon content does not have identical FTIR absorption peak, but its general absorption on the infrared light could be shown on a gradual absorption baseline reduction from 1300 cm^−1^ to 400 cm^−1^. This baseline reduction was only observed in the PCL/CS porous monolith with carbon content, indicating the presence of carbon content.

**Figure 7:**
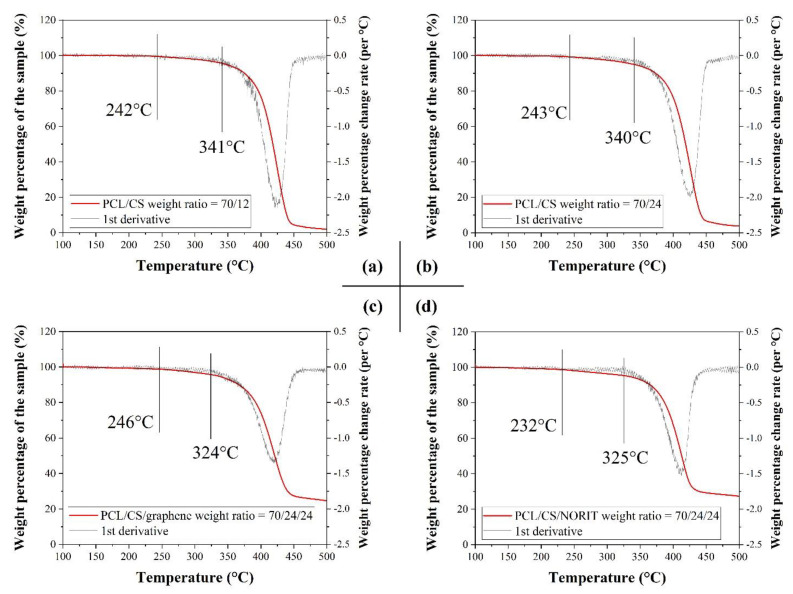
The TGA curves of the PCL/CS porous monoliths: (**a**) G-CS12; (**b**) G-CS24; (**c**) G-CS24-G; (**d**) G-CS24-N.

The residual content fraction and degradation temperature are also considered to validate the chitosan and carbon content in the PCL/CS porous monoliths. The PCL/CS porous monoliths with carbon content (Figure 7c,d) have a much higher residual weight at 500 °C than the monoliths with chitosan only (Figure 7a,b), indicating the presence of carbon content. Meanwhile, the degradation points of the PCL/CS monoliths could be found at around 240 °C and 340 °C (Figure 7a,b), validating the PCL and chitosan content in the monoliths by comparing it with the TGA curves of pure chitosan and PCL, which were degraded at 245 °C and 373 °C, respectively (Figure S1, Supplementary Data). The degrading point of PCL content was shifted to a lower temperature because formic acid is a degrading solvent for PCL, which reduced the molecular weight of the PCL molecules during the monolith fabrication. It was found that the addition of porous carbon content could further reduce the degrading temperature of PCL to around 325 °C (Figure 7c,d).

### 3.3. Porosity Analysis

The porosity of the PCL/CS porous monoliths was analysed by the BET method, while the half pore width distribution was modelled by the DFT method. The specific surface area and the average half pore width of the PCL/CS monoliths are listed in Table 2. The detailed half pore width distribution curves of the monoliths are included in Figure S2 (Supplementary Data). The addition of carbon helped to increase the overall porosity of the PCL/CS composite monoliths by introducing smaller pores within the monolith structure because there were two types of pores with 1.8 nm and 2.8 nm pore width found in the monoliths with carbon, while there was only one type of pore with a 2.6 to 2.8 nm pore width found in the monoliths without carbon content (Table 2).

### 3.4. Uraemic Toxin Absorption Performance

**Figure 8:**
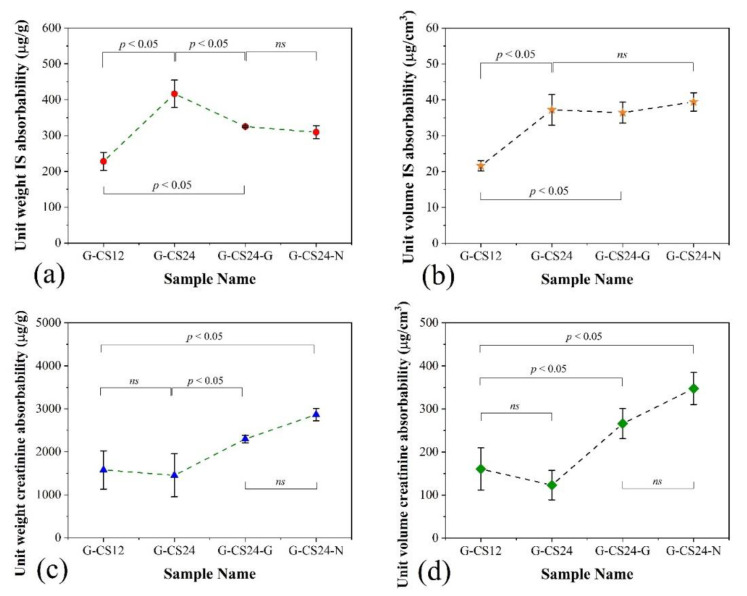
The comparison of unit (**a**,**c**) weight and (**b**,**d**) volume absorbability on the (**a**,**b**) albumin-bound indoxyl sulfate and (**c**,**d**) creatinine between the PCL/CS porous monoliths. (N = 3, ns = no significant difference).

The absorbability on creatinine and albumin-bound IS of the PCL/CS porous monoliths were tested and normalised with the weight and volume of the monoliths for comparison (Figure 8). The G-CS24 (PCL/CS weight ratio = 70/24) without carbon additives was found to have the best unit weight IS absorbability of all the PCL/CS porous monoliths (Figure 8a,b), while the unit volume IS absorbability of all CS24 porous monoliths had no significant difference, which indicated that the addition of porous carbon particles could not improve the albumin-bound IS absorption performance of the PCL/CS porous monolith, and the additional weight from these carbon particles would reduce the unit IS absorbability of the PCL/CS porous monoliths. On the other hand, carbon containing samples (G-CS24-G and G-CS24-N) had higher unit creatinine absorbability than the chitosan only samples (G-CS12 and G-CS24), indicating that the addition of porous carbon could enhance the creatinine absorption of the PCL/CS porous monoliths, while increasing the chitosan content in the composition failed to improve the creatinine absorption of the monoliths when comparing the unit creatinine absorbability of G-CS12 and G-CS24 samples (Figure 8a,b).

## 4. Discussion

Porous carbons have been reported to be adsorptive to small molecular water-soluble uraemic toxins, including creatinine, IS and hippuric acid due to their porous surface [10]. Previous studies of chitosan showed that it had limited capacity to absorb IS [14] with a non-physical equilibrium, and theoretically limited affinity for creatinine because of the repellent effect of the positive charges on the chitosan and creatinine molecules in aqueous environments [13]. Combining these two compounds with different absorptive characteristics in a stable PCL/CS monolith, could potentially expand the removable variety of uraemic toxins, so as to enhance the overall sorbent efficiency for dialysate regeneration for wearable artificial kidney devices.

The NORIT porous carbon powder was reported to have a higher creatinine (18,100 μg/g) and protein-bound indoxyl sulfate (3700 μg/g) adsorption capacity than the PCL/CS porous monolith in static tests [10], because the fine powder could have a better contact with the targeted toxin than the bulk PCL/CS monolith. However, the fine carbon powder could not be used directly in real dynamic dialysis practice because of the concerns of the leakage of carbon powder into the blood stream, which could be fatal. An absorbent courier is essential for real applications of these absorptive materials in powder form.

The G-CS24 without carbon additives had the highest IS absorbability by weight of all the PCL/CS porous monoliths, as the addition of porous carbon particles did not improve the albumin-bound IS absorption. However, carbon containing samples had greater absorption of creatinine compared to those only containing chitosan, indicating that the addition of porous carbon could enhance the creatinine absorption of the PCL/CS porous monoliths. Thus, we have shown that combining two different sorbents could function stably within the PCL/CS porous monolith, improving different uraemic toxin clearances, which demonstrates the potential application of this PCL/CS porous monolith as an absorbent courier for multiple absorptive agents.

Various absorptive materials have been developed for uraemic toxins clearance applying different principles and mechanisms, such as chitosan, zeolite, mesoporous carbon, and zirconium-based metal organic frameworks (Zr-based MOFs), which gives them different advantages on uraemic toxins removal. The combination of multiple absorbent materials within one absorbent courier matrix would be beneficial for practical uraemic toxins removal applications. The PCL/CS porous monolith structure could potentially provide a compatible and scalable composition environment with wide choice and high abundance of active additives for wearable artificial kidney absorbents or other liquid purification sorbent applications.

The dialysate generated in conventional haemodialysis (HD) is purified by nanofiltration technologies [20] for the regeneration of clean water. The waste generated by the PCL/CS porous monoliths after a single use is the monolith itself and the immobilized toxins inside. The PCL and chitosan are both thermally degradable according to the TGA curve of the PCL/CS monoliths. In order to treat the used PCL/CS monoliths, the used monoliths could be heated up to 500 °C where the PCL/CS monolith degrades completely (Figure 7), and treat those carbonised ash and undegraded residuals in the same way as the waste filtered from the HD dialysate [21,22].

## 5. Conclusions

The PCL/CS porous monoliths have been successfully fabricated using the vapour induced phase separation (VIPS) approach. Porous carbon was successfully incorporated into the PCL/CS porous monolith, forming an absorbent matrix with multiple absorptive contents for the removal of uraemic toxin (i.e., IS and creatinine). A maximum unit weight absorbability of 436 μg/g on albumin-bound indoxyl sulfate and 2865 μg/g on the creatinine has been achieved by the PCL/CS porous monoliths in a single-pass perfusion model within 1 h. This PCL/CS porous monolith configuration could offer advantages in easy fabrication, cost-effectiveness, formability, and wide composition allowance for multiple absorptive contents, which is beneficial for practical toxin clearance applications, such as wearable artificial kidneys.

## Figures and Tables

**Table 1:** The precursor solution list for the fabrication of PCL/CS porous monolith.

Abbreviation	PCL	Chitosan	Additives	Solvent Ratio (*v*/*v*)	Total Volume
G-CS0	0.7 g	0 g	No	DMF/formic acid = 7/3	10 mL
G-CS12	0.7 g	0.12 g	No	DMF/formic acid = 7/3	10 mL
G-CS24	0.7 g	0.24 g	No	DMF/formic acid = 7/3	10 mL
G-CS24-G	0.7 g	0.24 g	0.24 g graphene nanoplatelets	DMF/formic acid = 7/3	10 mL
G-CS24-N	0.7 g	0.24 g	0.24 g NORIT	DMF/formic acid = 7/3	10 mL

**Table 2:** The porosity analysis for the PCL/CS porous monoliths.

Sample ID	Specific Surface Area	Pore Types	Average Pore Width	
G-CS12	53.86 m^2^/g	Structure (s)		2.6 nm (s)
G-CS24	45.89 m^2^/g	Structure (s)		2.8 nm (s)
G-CS24-G	108.99 m^2^/g	Structure (s) + carbon (c)	1.8 nm (c)	2.8 nm (s)
G-CS24-N	80.17 m^2^/g	Structure (s) + carbon (c)	1.8 nm (c)	2.8 nm (s)

## Data Availability

The data presented in this study are available on request from the corresponding author.

## Supplementary Materials

The following are available online at https://www.mdpi.com/article/10.3390/nano11092247/s1, Figure S1: The TGA curve of pure chitosan and polycaprolactone (PCL), Figure S2: The half pore width distribution curve of the PCL/CS porous monoliths via DFT modelling: (**a**) G-CS12; (**b**) G-CS24; (**c**) G-CS24-G; (**d**) G-CS24-N.
