# Supplementary material for: Sponge-like Chitosan Based Porous Monolith for Uraemic Toxins Sorption
Source: Nanomaterials (Basel). 2021 Aug 30;11(9):2247. doi: 10.3390/nano11092247 (PMC8466498; doi:10.3390/nano11092247)
Supplement: Supplementary file 1 [file nanomaterials-11-02247-s001.zip › nanomaterials-1329406-supplementary.pdf]

Supplementary Materials

Sponge-like Chitosan Based Porous Monolith for Uraemic Toxins Sorption

Siyu Xiong [1], Yaxuan Lyu [2], Andrew Davenport [3] and Kwang Leong Choy [1,*]

[1] UCL Institute for Materials Discovery, University College London, London WC1E 7JE, UK; siyu.xiong.15@ucl.ac.uk
[2] MRC Laboratory for Molecular Cell Biology, University College London, London WC1E 6BT, UK; yaxuan.lyu.17@ucl.ac.uk
[3] UCL Centre for Nephrology, Royal Free Hospital, University College London, London NW3 2PF, UK; andrewdavenport@nhs.net
* Correspondence: k.choy@ucl.ac.uk

1. Characterization of Polycaprolactone/Chitosan Porous Monolith

1.1. TGA Analysis

The points of degradation of pure polycaprolactone (PCL) and pure chitosan (CS) used for the fabrication of PCL/CS porous monoliths were identified at 373 °C and 245 °C, respectively (Figure S1).

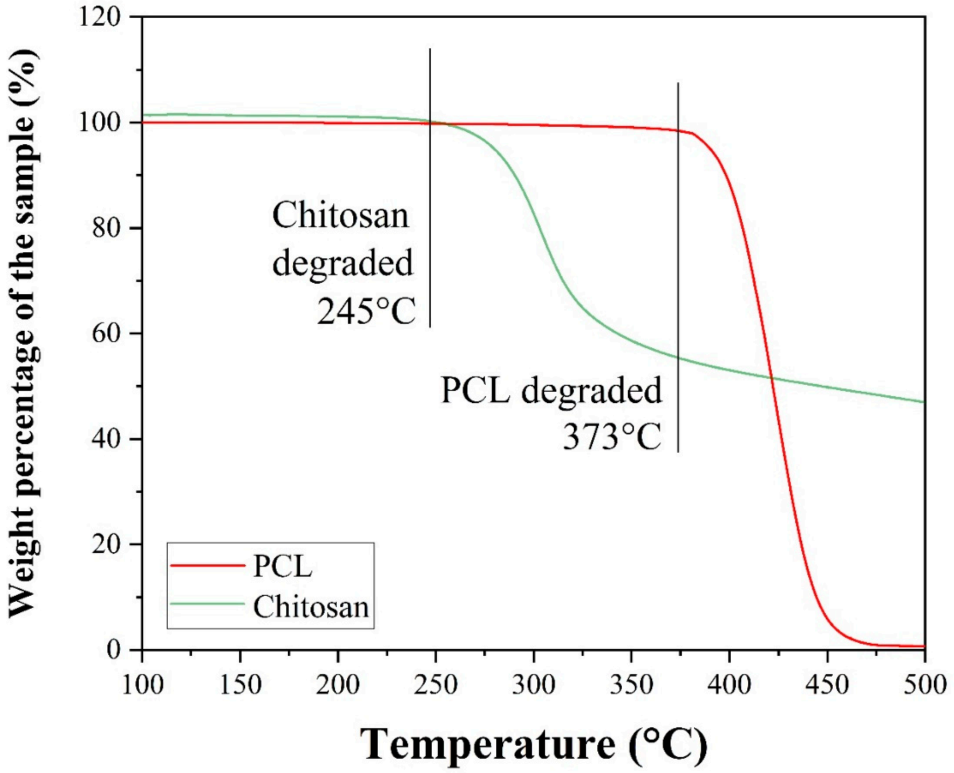

Figure S1. The TGA curve of pure chitosan and polycaprolactone (PCL).

1.2. Porosity Analysis

The half pore width distributions of the PCL/CS monoliths were modelled by the density-functional theory (DFT) method (Figure S2). It was found that there were two distinctive pore types: (1) structural pores resulted from cloth-like polymer layer folding, and (2) carbon surface pores. This pore distribution has indicated that the porous carbon particles were successfully distributed on the internal porous surface of the PCL/CS monolith.

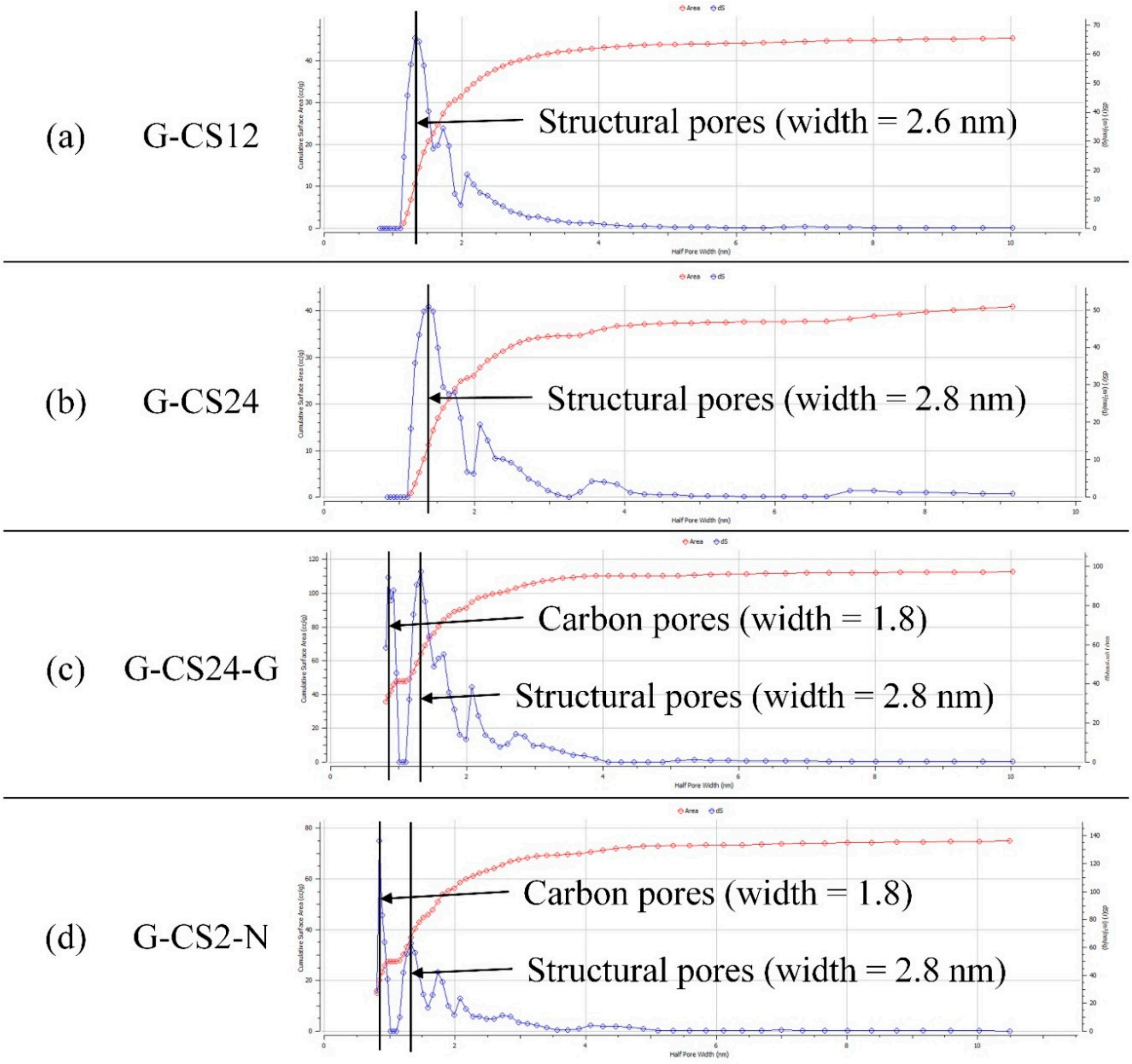

Figure S2. The half pore width distribution curve of the PCL/CS porous monoliths via DFT modelling: (**a**) G-CS12; (**b**) G-CS24; (**c**) G-CS24-G; (**d**) G-CS24-N.

Author Contributions: S.X.: Conceptualisation, Data curation, Formal analysis, Investigation, Methodology, Validation, Visualisation, Writing—original draft; Y.L.: Methodology, Visualisation; A.D.: Supervision, Writing—review and editing; K.L.C.: Conceptualisation, Funding acquisition, Methodology, Project administration, Resources, Supervision, Validation, Visualisation, Writing—review and editing. All authors have read and agreed to the published version of the manuscript.

Funding: This research was funded by St Peter's trust award 2016 (SPT 112) and UCL Therapeutic Acceleration Support (TAS) Fund 2018.

Institutional Review Board Statement: Not applicable.

Informed Consent Statement: Not applicable.

Acknowledgments: The partial financial support of Therapeutic Acceleration Support (TAS) Fund and St. Peter's Trust Fund are gratefully acknowledged.

Conflicts of Interest: The authors declare no conflict of interest.
